# Minimal residual disease guides postoperative therapy in a patient with ALK-rearranged adenocarcinoma: a case report

**DOI:** 10.3389/fonc.2026.1939564

**Published:** 2026-08-26

**Authors:** Xinghong Xian, Ke Wang, Liwen Wang, Hua Ke

**Affiliations:** 1 Department of Respiratory and Critical Care Medicine, West China Hospital of Sichuan University, Chengdu, China; 2 Department of Respiratory Medicine, Chengdu Seventh People's Hospital, Chengdu, China

**Keywords:** ALK rearrangement, brain metastasis, case report, ctDNA, minimal residual disease, non-small cell lung cancer

## Abstract

This case report explores the role of circulating tumor DNA (ctDNA)-based minimal residual disease (MRD) monitoring in predicting tumor recurrence or metastasis after consolidative surgery in a patient with ALK-rearranged non-small cell lung cancer (NSCLC). We present a case of a 43-year-old female patient with stage IVA ALK-rearranged NSCLC who initially responded to six months of Alectinib targeted therapy, underwent consolidative surgery with postoperative pathology confirming complete response in the resected primary tumor and lymph nodes, and continued two years of maintenance therapy. During this period, three consecutive MRD tests showed negative results, leading to discontinuation of postoperative therapy. However, two months after stopping Alectinib, the patient developed new brain metastasis in the occipital lobe, necessitating re-initiation of targeted treatment. This case demonstrates the current limitations of ctDNA-based MRD monitoring in reliably predicting recurrence and determining optimal treatment duration in NSCLC. Although MRD monitoring represents an important advancement in NSCLC management, our findings emphasize the need for additional research to validate its clinical applications, particularly in guiding treatment decisions and predicting disease recurrence. The results suggest that larger prospective studies are required to establish evidence-based protocols for MRD monitoring in NSCLC patients.

## Highlights

- ctDNA-based MRD monitoring failed to predict brain metastasis in an ALK+ NSCLC patient after therapy discontinuation.
- Further research is needed to validate MRD’s role in guiding treatment decisions for NSCLC.

## 1 Introduction

Lung cancer remains the leading cause of cancer-related mortality worldwide, with non-small cell lung cancer (NSCLC) accounting for approximately 85% of all cases1. Among NSCLC subtypes, 3-7% harbor ALK rearrangements, which are predominantly found in adenocarcinoma and are more common in younger patients, light or non-smokers, and those with lower body weight (1). Despite advances in targeted therapies, such as ALK inhibitors, the management of ALK-positive NSCLC remains challenging, particularly in predicting and preventing disease recurrence.

Minimal residual disease (MRD) refers to the presence of tumor-derived molecular abnormalities detected through liquid biopsy (e.g., circulating tumor DNA [ctDNA]) after treatment, which are undetectable by conventional imaging modalities such as PET/CT or standard laboratory tests (2). MRD reflects the persistence of residual tumor cells and is strongly associated with an increased risk of recurrence and poorer survival outcomes. Studies have consistently demonstrated that MRD-positive patients exhibit significantly higher recurrence rates and lower survival rates compared to MRD-negative patients following surgery (2).

Current expert consensus suggests that de-escalation of postoperative therapy may be considered in patients with negative postoperative MRD (3). However, there is a lack of prospective clinical trial data to guide the timing and strategy of de-escalation of postoperative therapy, leaving this approach largely investigational.

In this report, we present a case of a patient with ALK-rearranged adenocarcinoma who underwent consolidative surgery, MRD monitoring, and de-escalation of targeted therapy, but subsequently experienced disease recurrence. This case highlights the potential limitations of MRD monitoring in guiding treatment decisions and underscores the need for further research to optimize its clinical application in NSCLC.

## 2 Case presentation

### 2.1 Clinical history and diagnostic evaluation

**Figure 1:**
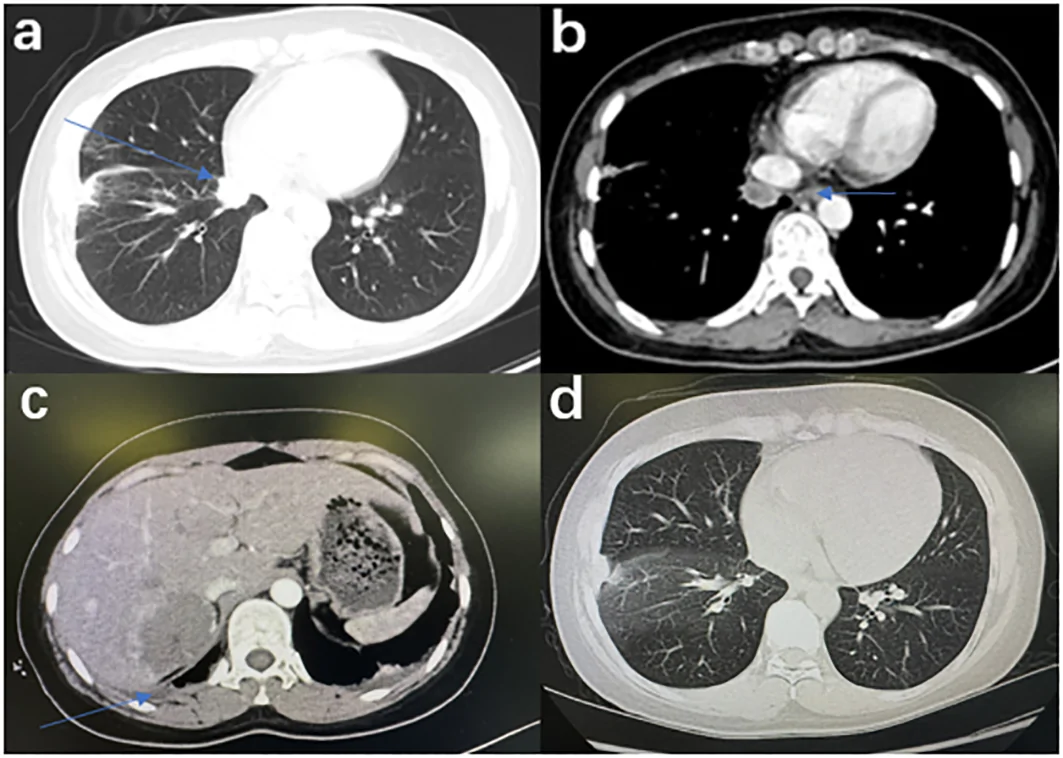
Initial and follow-up chest CT findings. **(a)** Plain CT scan (THK: 2 mm) showing a soft tissue nodule (arrow) measuring approximately 2.0 × 1.7 cm near the hilum of the right lower lung. **(b)** Contrast-enhanced CT scan (THK: 5 mm) demonstrating enlargement of right hilar and mediastinal lymph nodes (arrowheads). **(c)** Contrast-enhanced CT showing bilateral pleural effusion (arrows). **(d)** Follow-up contrast-enhanced CT after six months of alectinib therapy demonstrating partial response.

A 43-year-old female patient was admitted in May 2021. Initial chest CT revealed a 2.0 × 1.7 cm soft tissue nodule near the hilum of the right lower lung, accompanied by right hilar and mediastinal lymph node enlargement, and bilateral pleural effusion (**Figure 1**). Tumor marker analysis showed an elevated level of CA-125 (86.90 U/ml) in the blood. Pleural fluid analysis further revealed elevated levels of CEA (8.7 ng/ml), CA-125 (1356 U/ml), CYFRA21-1 (17.3 ng/ml), and SCC (9.74 ng/ml). Cytological examination of the pleural fluid confirmed the presence of adenocarcinoma cells. No remarkable positive signs were found on physical examination. The patient had no smoking history and reported no family history of malignancy. Contrast-enhanced CT of the chest and upper abdomen, SPECT bone scan, and brain MRI showed no evidence of distant metastasis. Next-generation sequencing (NGS) identified an EML4-ALK (E6:A20) gene rearrangement with a fusion abundance of 5.8%.

Based on the clinical and diagnostic findings, The patient was diagnosed with right lower lung adenocarcinoma, cT1N2M1a, Stage IVA (8th edition TNM classification), and EML4-ALK-positive.

### 2.2 Treatment and follow-up

In July 2021, she was initiated on oral Alectinib (600 mg twice daily). MRD monitoring was performed every six months using a tumor-informed ctDNA detection platform (3DMed, Shanghai, China). The custom panel was designed based on the patient’s baseline tumor tissue NGS results, targeting the EML4-ALK fusion junction and other somatic variants. The assay employed a mean sequencing depth of >30,000×, with a limit of detection (LOD) of 0.004% mutant allele frequency (MAF), as validated in the manufacturer’s analytical studies. Comparable tumor-informed MRD assays have reported equivalent sensitivity (0.004%) in peer-reviewed NSCLC studies (17). Each MRD test required 10 mL of peripheral blood, from which plasma was isolated for ctDNA extraction and sequencing. By August 2021, a partial response (PR) was achieved. Following sustained PR, the patient underwent video-assisted thoracoscopic surgery (VATS) in January 2022, which included right lower lobectomy, systematic lymph node dissection, lysis of pleural adhesions, and ablation of pleural nodules. Postoperative pathology of the resected specimens (right lower lobe and lymph nodes) confirmed a complete pathologic response, with no residual tumor tissue (pT0N0Mx) and negative bronchial margins. However, the pleural (M1a) component was managed by ablation without histologic confirmation; therefore, pathologic complete response could not be definitively established for the pleural disease. The absence of pleural residual disease was presumed based on intraoperative findings and postoperative imaging.

Given the patient’s initial Stage IV diagnosis, she continued Alectinib (600 mg twice daily) as postoperative therapy, with MRD monitoring performed every six months. The patient showed good adherence to Alectinib therapy throughout the postoperative treatment period, with no dose interruptions or discontinuations due to adverse events. After two years of postoperative therapy and three consecutive negative MRD tests, Chest and Abdominal CT, brain MRI, and Bone Scan in January 2024 showed no evidence of tumor recurrence.

### 2.3 Outcome and disease recurrence

**Figure 2:**
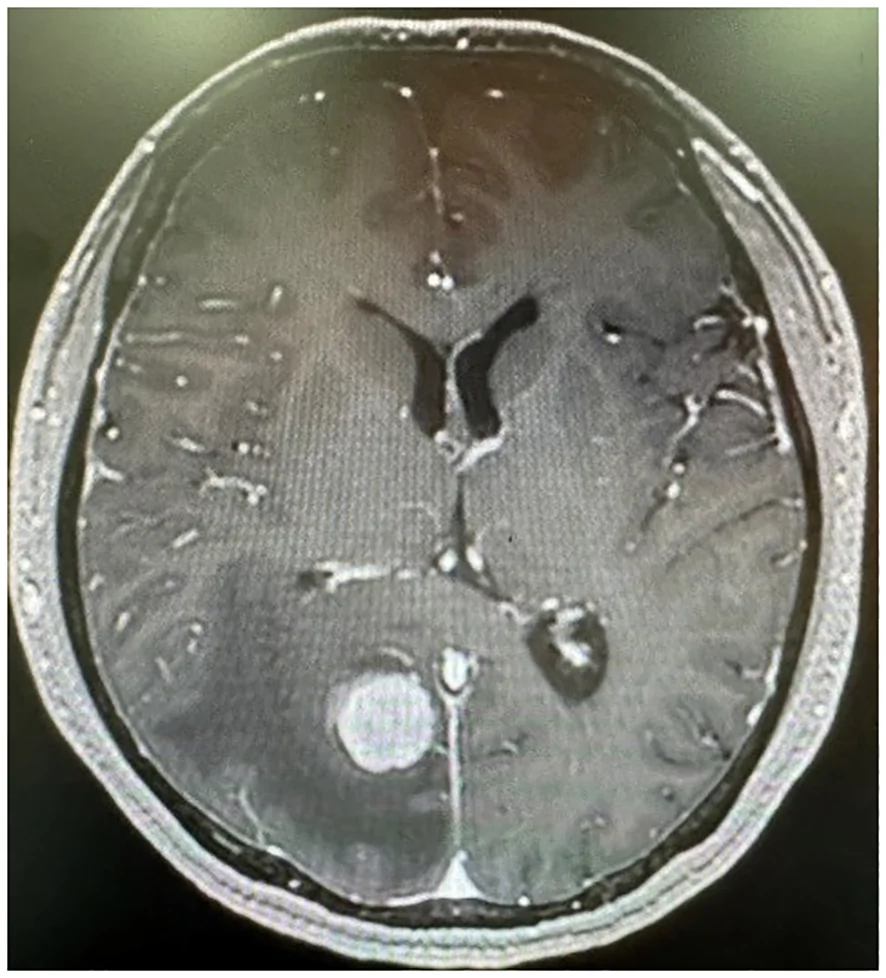
Brain MRI from March 2024 (THK:1.6mm). A new metastatic lesion, measuring approximately 2.3 × 2.0 cm, was identified in the occipital lobe, demonstrating moderate heterogeneous enhancement and surrounding edema.

Two months after discontinuing Alectinib, the patient developed dizziness. Follow-up brain MRI revealed a new metastatic lesion in the occipital lobe (**Figure 2**). No tumor recurrence was detected on chest or abdominal CT. Alectinib therapy was promptly resumed.

Of note, ctDNA-based MRD testing was not repeated at the time of brain metastasis diagnosis, as the patient required immediate clinical management for acute neurological symptoms.

**Figure 3:**
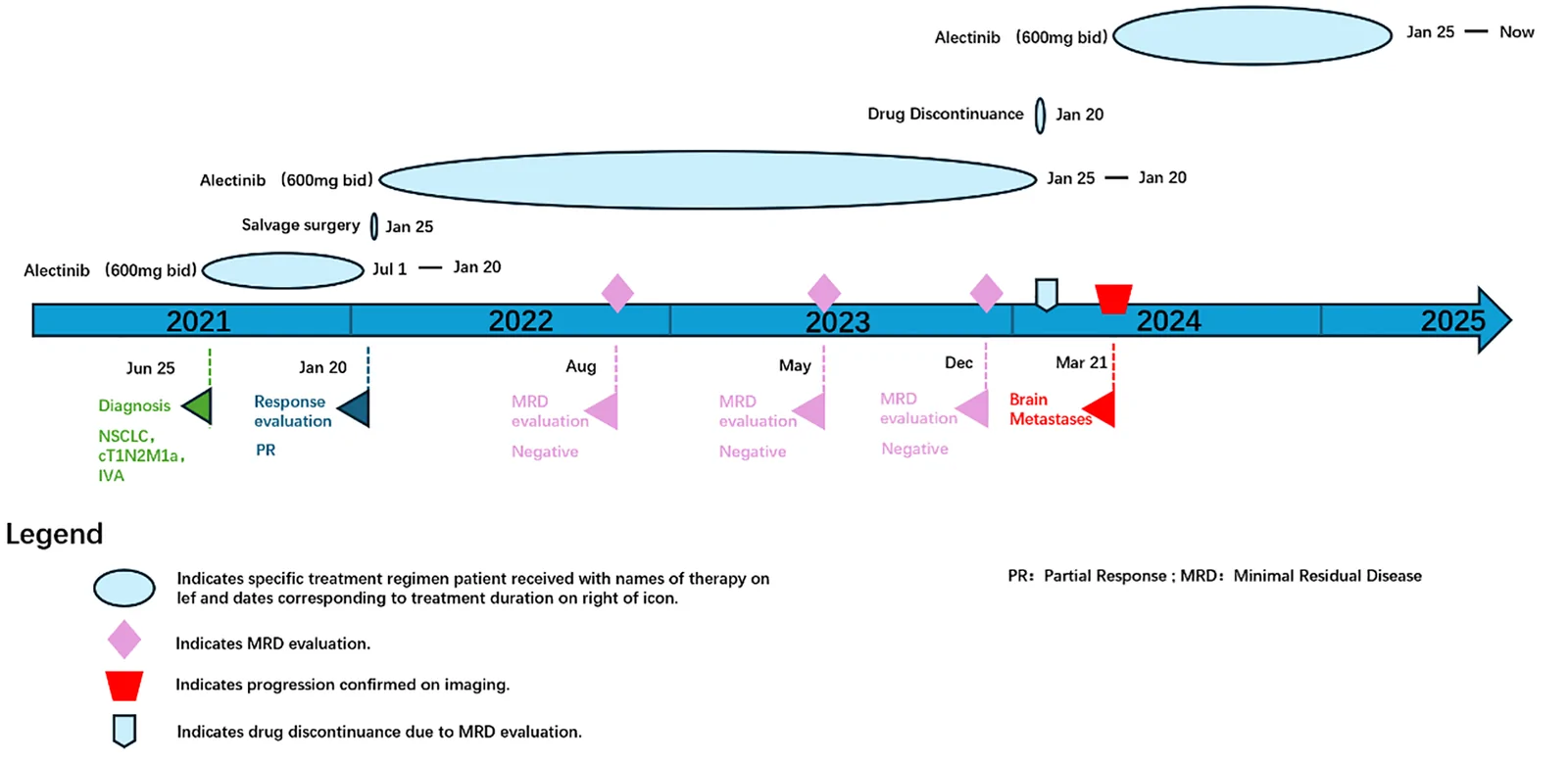
Patient’s treatment timeline.

The timeline of the patient’s treatment course is summarized in **Figure 3**.

## 3 Discussion

We present a case of stage IVA ALK-rearranged NSCLC in which isolated brain metastasis occurred two months after discontinuation of Alectinib, despite two years of negative peripheral ctDNA MRD monitoring following consolidative surgery. In the international multicenter Phase III ALINA trial, the postoperative use of Alectinib in completely resected ALK-positive NSCLC demonstrated significant clinical benefits (4). Alectinib reduced the risk of disease recurrence or death by 76% compared to platinum-based chemotherapy (HR = 0.24, 95% CI: 0.13-0.43, P<0.001). The three-year disease-free survival (DFS) rate was 88.7% in the Alectinib group, significantly higher than the 54.0% observed in the chemotherapy group (4). Additionally, Alectinib markedly reduced the risk of central nervous system (CNS) metastasis, with a three-year CNS recurrence-free rate of 95.5% compared to 79.7% in the chemotherapy group (4). It is important to acknowledge that the ALINA trial enrolled patients with completely resected stage IB–IIIA ALK-positive NSCLC, whereas the patient in the present case was initially diagnosed with stage IVA disease and underwent consolidative surgery following systemic targeted therapy. These clinical contexts are fundamentally different. Therefore, the compelling evidence from the ALINA trial cannot be directly extrapolated to justify postoperative therapy in this setting. Rather, our decision to continue alectinib postoperatively was based on the recognition that the patient had achieved a pathologic complete response after consolidative surgery, and that consolidative local therapy combined with continued targeted therapy is a recognized strategy for selected patients with oligometastatic or oligoresidual disease following effective systemic treatment. This approach was adopted after multidisciplinary team discussion and thorough shared decision-making with the patient, with the understanding that the optimal duration of postoperative targeted therapy in this unique population remains undefined and requires further investigation. MRD refers to molecular abnormalities of cancerous origin that are detected through liquid biopsy after treatment and are not evident using conventional imaging or laboratory methods (5). MRD signifies the persistence of lung cancer and the potential for clinical progression. Initially conceptualized in the context of hematologic malignancies, MRD has recently been applied to solid tumors, including lung cancer, for predicting recurrence. Current studies demonstrate that MRD assessment based on circulating tumor DNA (ctDNA) exhibits higher sensitivity and specificity for predicting tumor recurrence compared to traditional clinical or imaging methods (6). Furthermore, combining ctDNA analysis with imaging can detect potential NSCLC progression more promptly than imaging alone (7). Some studies have also indicated that early-stage lung cancer detection guided by ctDNA analysis improves patient outcomes (8).

Despite aggressive curative treatments for NSCLC, the risk of tumor recurrence remains high (9). Therefore, MRD monitoring plays a critical role in identifying patients at higher risk of recurrence. Postoperative ctDNA detection has been shown to effectively assess recurrence risk in clinical studies. Patients with MRD positivity after surgery exhibit a higher recurrence rate and lower survival rate compared to those with MRD negativity. MRD detection can predict tumor recurrence months earlier than clinical imaging, providing valuable guidance for individualized follow-up and interventional treatment. Single-center randomized controlled trials (RCTs) have demonstrated that ctDNA monitoring may guide de-escalation therapy in patients with advanced NSCLC. Notably, there was no significant difference in progression-free survival (PFS) between patients who resumed treatment based on ctDNA elevation prior to imaging progression and those who continued long-term drug therapy, with PFS of 20.3 months versus 20.2 months, respectively (10).

Currently, several clinical trials are underway to further validate the significance of postoperative MRD monitoring in the treatment of NSCLC. Expert consensus recommends considering de-escalation therapy in cases where postoperative MRD status is negative (3). However, there is currently a lack of prospective clinical trial data to guide the optimal timing and approach for de-escalation therapy.

Although numerous studies have highlighted the value of postoperative MRD monitoring in predicting tumor recurrence or distant metastasis in NSCLC (10–12), this case report presents a unique scenario. The patient developed brain metastasis two months after discontinuing targeted therapy (Alectinib), despite two years of postoperative MRD monitoring with three consecutive negative results.

A review of the literature suggests the following potential reasons for the observed outcomes: 1. Genetic Phenotypes Influence Detection Rates: Different genetic phenotypes of NSCLC can significantly impact the detection rate of circulating ctDNA. A pan-cancer ctDNA study revealed that the ctDNA detection rate in NSCLC was 63.4% for Stages I-III and 79.7% for Stage IV (13). Specific genetic mutations, such as TP53 and EGFR, have been shown to increase ctDNA detection rates (14). Notably, TP53 mutations independently predict higher ctDNA levels, potentially reflecting increased tumor cell turnover or metabolic activity. 2. Complex Tumor Mutation Backgrounds Demand Advanced Detection Methods: Approximately 70% of lung adenocarcinomas harbor at least one targetable driver gene mutation, such as EGFR, ALK, or ROS1 (15). These mutations include point mutations, insertions, deletions, and fusions, creating a complex mutation landscape that necessitates more sensitive MRD detection technologies. 3. Evolving ctDNA-Based MRD Detection Technologies: Current MRD detection platforms for lung cancer, such as CAPP-Seq, Signatera, and ArcherDx (used in the TRACERx study (6)), are continuously advancing. CAPP-Seq, a next-generation sequencing (NGS) technology, utilizes customized capture probes targeting common lung cancer mutations, enabling efficient mutation detection within a minimal genomic region (8). While CAPP-Seq is highly sensitive and widely used for early cancer monitoring and postoperative MRD detection, it has limitations. First, it requires prior knowledge of the tumor’s mutation profile, which can be challenging if tumor tissue samples are unavailable. Second, its minimum detectable sensitivity is approximately 0.004% mutant allele frequency (MAF) (8), necessitating large plasma samples with extremely low ctDNA concentrations. Furthermore, even more sensitive MRD technologies have recently emerged, achieving detection limits as low as approximately 0.0001% variant allele frequency through approaches such as phased variant enrichment sequencing (PhasED-seq). Whether such ultra-sensitive methods would have detected residual disease in this patient remains speculative, but they may further improve MRD sensitivity for intracranial disease surveillance in future applications (16). 4. Challenges in Reflecting Intracranial MRD Status: Current MRD detection relies on peripheral blood samples, which may not accurately reflect intracranial MRD status (6). This limitation poses risks when de-escalating therapy based on MRD results, as it may fail to detect brain-specific residual disease.

Beyond the blood-brain barrier limitation, other potential explanations for this discordance merit consideration. First, stochastic sampling limitations inherent to a single 10 mL peripheral blood draw at very low tumor burden may have contributed to false-negative results. Second, the possibility of *de novo* CNS-restricted clonal seeding cannot be excluded—the brain metastasis may have arisen from a subclone that either did not shed ctDNA into the peripheral circulation or shed variants not captured by the tumor-informed panel. These alternative mechanisms highlight the complexity of MRD interpretation in patients with CNS-only relapse.

A key limitation of this case is that ctDNA was not re-tested at the time of brain metastasis diagnosis. Such a measurement would have helped distinguish between a true CNS “blind spot” and a lead-time effect. This underscores the need for future studies to incorporate paired peripheral and cerebrospinal fluid ctDNA analysis at the time of suspected CNS relapse.

In summary, the clinical application of ctDNA-based MRD detection for predicting postoperative tumor recurrence or metastasis in NSCLC has limitations. MRD results should be interpreted in conjunction with traditional imaging and pathological indicators to comprehensively assess the risk of recurrence or metastasis.

## 4 Conclusion

This case report highlights both the potential and limitations of ctDNA-based MRD monitoring in guiding postoperative therapy for ALK-rearranged NSCLC. While the patient achieved a complete pathologic response in the resected specimens after consolidative surgery and maintained negative MRD results for two years, the rapid development of brain metastasis following therapy discontinuation underscores the challenges in relying solely on MRD to predict recurrence. The findings suggest that current MRD technologies may have limitations in detecting residual disease, particularly in the central nervous system, and emphasize the need for integrating MRD monitoring with traditional imaging and clinical assessments. Further research, including larger prospective studies, is essential to validate the clinical utility of MRD and establish evidence-based guidelines for treatment de-escalation in NSCLC. Until then, caution should be exercised when making therapeutic decisions based on MRD results alone.

## 5 Patient perspective

The patient was informed about the rationale for MRD monitoring and the potential risks and benefits of treatment de-escalation. She expressed understanding and agreed to the decision to discontinue alectinib after two years of negative MRD results. When dizziness developed and brain metastasis was diagnosed, she experienced anxiety but remained trustful of the treatment team. She was promptly restarted on alectinib and has been closely followed up. She provided written informed consent for the publication of this case report and hopes that sharing her experience may help other patients and physicians make more informed decisions.

## Data Availability

The original contributions presented in the study are included in the article/supplementary material. Further inquiries can be directed to the corresponding author.
